# Extreme Low-Temperature Stress Affects Nutritional Quality of Amino Acids in Rice

**DOI:** 10.3389/fpls.2022.905348

**Published:** 2022-06-02

**Authors:** Min Kang, Gurong Liu, Yaowen Zeng, Jia Zhou, Jiangyi Shi, Liang Tang, Leilei Liu, Weixing Cao, Yan Zhu, Bing Liu

**Affiliations:** Key Laboratory for Crop System Analysis and Decision Making, Jiangsu Key Laboratory for Information Agriculture, National Engineering and Technology Center for Information Agriculture, Engineering Research Center of Smart Agriculture, Jiangsu Collaborative Innovation Center for Modern Crop Production, Ministry of Education, Ministry of Agriculture, Nanjing Agricultural University, Nanjing, China

**Keywords:** rice, low-temperature stress, flowering stage, grain filling stage, amino acid, climate change

## Abstract

Global climate change has increased the frequency of extreme climate events, and their effects on the nutritional quality, especially on amino acids in rice, have not been quantified. The data from a 3-year low temperature stress (LTS) experiment including two rice varieties (Huaidao 5 and Nanjing 46), seven minimum/maximum temperature levels (one optimal 21/27°C and six LTS levels from 17/23 to 6/12°C), and three LTS durations (3, 6, and 9 days) after flowering, revealed significant interactive effects of LTS at different stages, durations, and temperature levels on the content and accumulation of amino acids. LTS increased rice total amino acid content, while decreasing its accumulation, with higher sensitivities to LTS at the flowering stage than at the grain filling stage. In most treatments, the lysine (the first limiting amino acid) and phenylalanine content were increased under LTS at early and peak flowering stages but decreased at the grain filling stage in both varieties, and only leucine content was increased at all three stages after flowering, while the content of other essential amino acids differed among the two varieties. With an increase of 1°C·d per day in the accumulated cold degree days, the relative content of the essential amino acids was increased by 0.01–0.41%, depending on the rice variety and growth stage. Our results suggest that LTS can improve nutritional quality of amino acids of rice grains in terms of amino acids content, especially at flowering stage. These results provide critical insights for assessing the potential impact of extreme climates on the nutrient quality of rice under future climate change.

## Introduction

Rice is one of the three staple foods for more than half of the world’s population. Nowadays, improved awareness and living standards have increased the demand for better rice quality (Zhu et al., 2020), with emphasis on rice protein and amino acids, as well as the composition and balance of essential amino acids, as important indicators for measuring rice nutritional quality (Ma et al., 2020). Amino acids are not only essential compounds in plants but are also the main constituents of proteins and signal molecules in organisms (Guo et al., 2021). Some of them, with pharmacological effects, can prevent coronary heart disease, relieve mental stress, and determine the nutritional value of rice (Huang et al., 2019a). According to FAO, the number of people suffering from chronic malnutrition caused by insufficient intake of amino acids reached 515 million worldwide in 2017. And the people were mainly in developing countries and regions where rice is the staple food and amino acids in rice grains, especially essential amino acids (Wong et al., 2015; Kline et al., 2016). Rice production may be threatened by climate change in the future, especially by extreme climate events (Sanchez et al., 2014; Hasegawa et al., 2021), and the occurrence of extreme low-temperature stress (LTS) after flowering will notably affect rice quality (Dingkuhn et al., 2015; Jia et al., 2019). Thus, it is important to evaluate and quantify the impact of extreme LTS on the accumulation of amino acids in rice grains.

Temperature is one of the main factors significantly affect the quality, especially during critical growth stages (Zhong et al., 2005). Among the different growth stages, rice grain quality was found to be the most sensitive to temperature stress during the flowering and grain-filling stages. Consequently, rice yield and quality may be affected when the temperature is too low (Sanchez et al., 2014). With the increasing demand for food, rice-growing areas are expanding to higher altitudes and latitudes, and thus, the probability of LTS influencing rice quality is also gradually increasing (Liang et al., 2021). Rice production in approximately 24 countries worldwide, including major rice-producing countries such as China and Japan, has already encountered notable LTS (Zhang et al., 2017). Moreover, the frequency and severity of extreme temperature events are increasing even under global warming due to phenological shifting, and the frequency of LTS is higher at the flowering and grain filling stages of late rice and single rice. In recent years, most studies have focused on the impact of climate changes on rice grain yield (Shi et al., 2021), the majority of which are related to high-temperature stress and drought (Chen et al., 2017; Zhen et al., 2020). A few studies have focused on the impact of LTS on rice quality based on protein and starch content (Zeng et al., 2016). These studies indicate that extreme LTS can significantly decrease grain appearance and cooking quality (Siddik et al., 2019) but can increase protein and amylose accumulation (Hirano and Sano, 1998). A few studies have focused on the effects of LTS after flowering on the composition of amino acids in rice grains. In addition, most relevant studies only involved a specific stage or a single duration of LTS (Huang et al., 2019a), ignoring the effects of different low-temperature intensities and durations on rice quality. Therefore, further studies are needed to explore the effects of LTS intensities and durations at different critical post-anthesis stages on the composition of amino acids in rice. At the same time, most projections of climate change impacts have focused on food security (Liu et al., 2018). However, the impact of extreme climate events on nutritional security has generally been ignored, particularly in developing countries.

In the present study, two rice varieties with different LTS tolerance were used to conduct low-temperature treatments with different intensities and durations in phytotrons during the flowering and grain filling stages to investigate the effects of LTS after flowering stage on amino acids in rice grains. We aimed to (1) determine the effects of LTS with different intensities and durations on the nutritional quality of amino acids in rice grains during the flowering and grain filling stages, and (2) quantify the relationship between LTS and the nutritional quality of amino acids in rice grains. The study results can provide important insights for the quantitative simulation and prediction of the nutritional quality of rice grains under future climate change scenarios.

## Materials and Methods

### Experimental Design

Environment-controlled phytotron experiments with two rice varieties, namely, Huaidao 5 and Nanjing 46, were conducted from 2018 to 2020 at Rugao Base (32°16N, 120°45E) in Jiangsu Province, China. Under LTS, the Nanjing 46 cultivar showed a larger reduction in grain yield compared to the Huaidao5 cultivar., indicating that Nanjing 46 was more sensitive to LTS than Huaidao 5 (Ali et al., 2021). Seeds were sown in May in a nearby field (Table 1) and raised on a dry seedbed. The 3-leaves old seedlings were transplanted into plastic pots (height 35.6 cm, inner diameter 29.8 cm, volume 25.0 l, filled with 22 kg of soil) with two plants per hill and six plants per pot. The pots were placed close together at a density of approximately 11 pots per m^2^ (equivalent to 66 plants per m^2^), which is similar to the planting density of typical japonica rice grown in the local rice cropping system. Next, 1.5 g N, 1.5 g P_2_O_5_ and 2 g K_2_O were applied in each pot as basal fertilizer before transplanting, and an additional 0.3 g N and 1.2 g N were top-dressed at mid-tillering and panicle initiation, respectively. Weed, disease, and pest control were conducted according to the local standards of rice cultivation to avoid biotic and abiotic stresses. Rice plants in pots were grown under ambient conditions prior to the LTS treatments. When they reached the target developmental stages of flowering and grain filling, pots at similar growth stages and having the same number of panicles were transferred into phytotrons for the LTS treatments. After the treatments were completed, the rice plants were randomly divided into three replicates and moved out to grow in an ambient environment until harvest.

**Table 1:** Summary of the post-heading low-temperature stress treatments.

Year	Cultivar	Treatment stage	Temperature levels (T min/T max; °C); Duration (days)	Sowing date (mm/dd)	Transplanting date (mm/dd)
2018	Huaidao 5	S1, S2, S3	T1 (21/27); D1 (3), D2 (6), D3 (9)	05/14	06/13
	Nanjing 46		T2 (17/23); D1 (3), D2 (6), D3 (9)	05/18	06/17
2019	Huaidao 5		T3 (13/19); D1 (3), D2 (6), D3 (9)	05/24	06/24
	Nanjing 46		T4 (9/15); D1 (3), D2 (6), D3 (9)	05/29	06/26
2020	Huaidao 5	S1, S2, S3	T1 (21/27); D1 (3), D2 (6), D3 (9)	05/16	06/13
			T2* (16/22); D1 (3), D2 (6), D3 (9)		
	Nanjing 46		T3* (11/17); D1 (3), D2 (6), D3 (9)	05/16	06/18
			T4* (6/12); D1 (3), D2 (6), D3 (9)		

*S1, S2, and S3 represents the early stage of flowering (50% of panicles were flowering), peak stage of flowering (3 days after the early stage of flowering), and early stage of grain filling (12–15 days after flowering), respectively*.

In this study, the early stage of flowering (50% of panicles flowering, S1), the peak stage of flowering (3 days after the early stage of flowering, S2), and the early stage of grain filling (12–15 days after flowering, S3) were the target stages for low-temperature treatments. According to previous studies, the lower threshold temperature of japonica rice was 14 ± 2°C at the flowering stage and 17.9 ± 2.3°C at the filling stage (Sanchez et al., 2014). Thus, seven temperature levels with minimum/maximum temperature of 21/27°C (T1, CK), 17/23°C (T2), 16/22°C (T2^*^), 13/19°C (T3), 11/17°C (T3^*^), 9/15°C (T4), and 6/12°C (T4^*^) were tested at three temperature durations of 3 days (D1), 6 days (D2), and 9 days (D3); the combination of temperature level and duration regimes over 2018–2020 is summarized in Table 1. T1 was considered the optimal temperature level for rice growth, while T2, T2^*^, T3, T3^*^, T4, and T4^*^ were considered LTS treatments.

**Figure 1:**
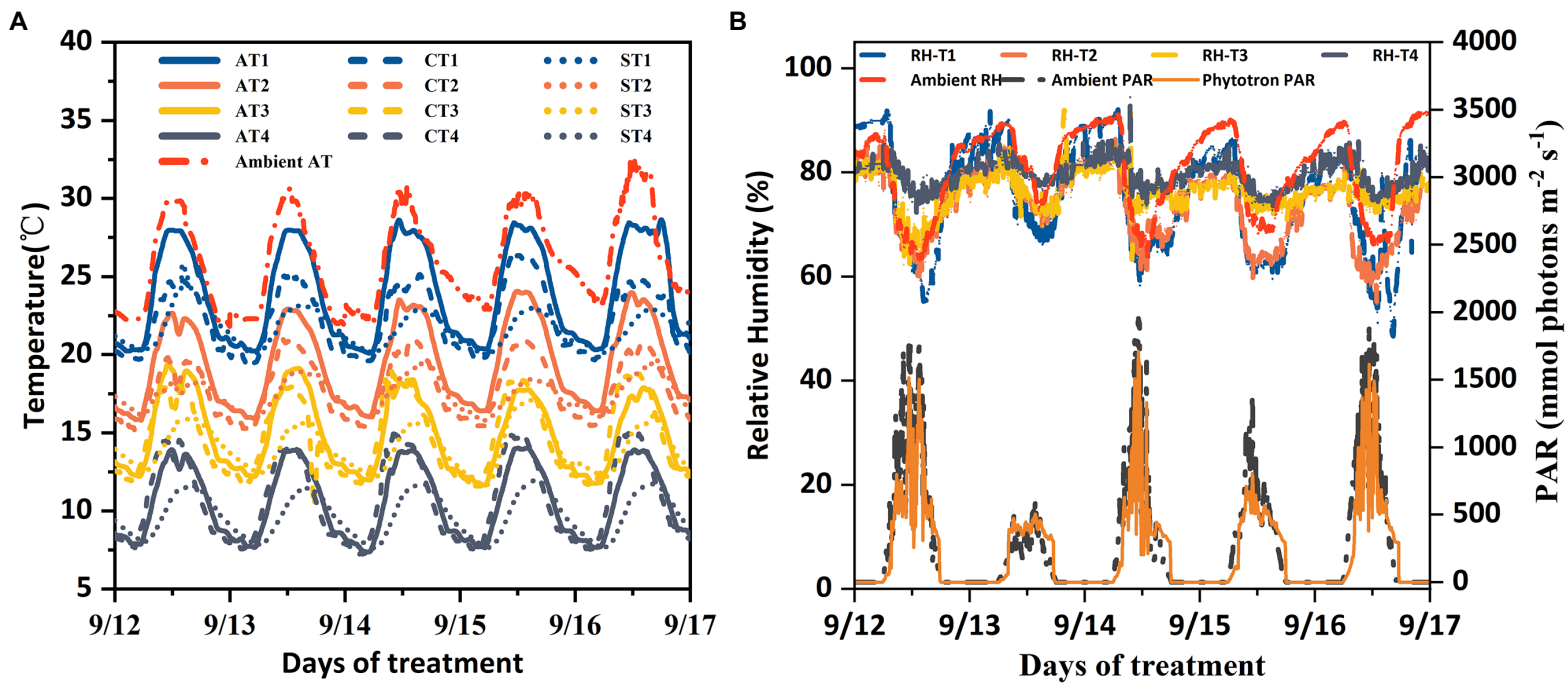
Diurnal changes of four levels of air temperature (AT; **A**, solid lines), canopy temperature (CT; **A**, broken lines), soil temperature (ST; **A**, dotted lines), relative humidity (RH; **B**, broken lines), and incident photosynthetically active radiations (PAR; **B**, solid line) measured in low-temperature stress treatment chambers in 2018. Lines with double dots show the ambient AT, PAR, and RH of the same days.

Four phytotrons (3.4 m × 3.2 m × 2.8 m) made with high-transparency glass were used for low-temperature treatments. Each phytotron was installed with an automatic equipment to precisely control the temperature, humidity, light, and CO_2_ concentration, simulating the pattern of the local ambient environment. The temperature was controlled within an accuracy of 1°C across all four phytotrons. Relative humidity and CO_2_ concentration ranged from 20 to 95% and 350–2,000 μmol·mol^−1^, respectively. Halogen lamps were used to supplement the light to ensure light intensity same as the local ambient environment in each phytotron (Figure 1B). Air temperature and humidity, soil temperature and moisture, and photosynthetically active radiation in the phytotrons were measured with VP-3 sensor (Decagon Devices, Pullman, WA, United States), 5TM sensor (Decagon Devices, Pullman, WA, United States), and PYR solar radiation sensor (Decagon Devices, Pullman, WA, United States), respectively. All data were recorded and transmitted wirelessly every 5 min using an EM50G (Decagon Devices, Pullman, WA, United States). Canopy temperature (*T*_canopy_) was measured using an infrared radiometer SI-111 (Apogee Instruments, Logan, UT, United States), installed 1.6 m above the ground and facing diagonally downward toward the plant surface for a sampling area of 0.8 m^2^. Daily temperature variation in the phytotrons was controlled continuously to simulate the natural environment. The measured diurnal variations in air and canopy temperature in the phytotrons during the treatment period are shown in Figure 1, which conforms to that under natural conditions. This ensured that the effects of the LTS during the investigation were similar to those under field conditions.

### Sampling and Measurements

#### Sampling and Harvest

After LTS treatment, all pots were moved to the external natural environment. The maturity date was determined when the color of more than 80% of grains turn into yellow, all glumes and stems became yellow and only the first and second internode remained slightly green. LTS delayed the phenology of rice and the detailed days from sowing to maturity for each treatment was shown in Supplementary Figure S1. At maturity, six pots were randomly selected from each treatment for sampling and dried in the natural environment to investigate yield and yield-contributing traits, such as 1,000-grain weight and the number of panicles and grains per spike. Then, the grains were husked and got the brown rice yield. Then the brown rice was ground into powder to analyze the grain quality parameters.

#### Amino Acid Content and Accumulation

Using an amino acid analyzer (S-433D, SYKAM, Germany), the composition and proportion of amino acids were analyzed. Approximately 2.0 g (accurate to 0.0001 g) of rice grain samples were degreased and transferred into tubes containing 10 ml of 6 mol L^−1^ HCl and hydrolyzed at 110°C for 22 h. The hydrolyzed solution was filtered, and 1.0 ml filtrate was accurately drawn using a test tube concentrator and dried under reduced pressure at a temperature of 40–50°C. Finally, a known volume (1 ml) of the supernatant was injected into an amino acid analyzer to estimate the amino acids composition of each sample. A total of 17 amino acids, including seven essential amino acids [valine (Val), threonine (Thr), methionine (Met), isoleucine (Ile), leucine (Leu), phenylalanine (Phe)] and ten non-essential amino acids [lysine (Lys), serine (Ser), glutamate (Glu), glycine (Gly), alanine (Ala), cysteine (Cys), tyrosine (Tyr), histidine (His), arginine (Arg), proline (Pro), and aspartic acid (Asp)] were estimated. The accumulation of amino acids was calculated by multiplying the amino acid content by the grain yield of brown rice.

#### Quantifying the Relationship Between LTS and Nutrimental Quality of Amino Acids

The impact of LTS on the nutritional quality of amino acids was quantified by the linear regression method. The accumulated cold degree days (ACDD, °C·d), defined as the accumulated temperature below the critical temperature threshold, has been widely used to quantify the comprehensive effects of low-temperature intensities and durations on grain yield and biomass accumulation in rice, wheat, and other crops (Shimono et al., 2007). In this study, ACDD was used to quantify the comprehensive effects of low-temperature intensity and duration on the nutritional quality of rice grains. ACDD was calculated according to the following formulas:


(1) $$\mathrm{ACDD}=\overset{m}{\underset{i=1}{\sum }}{\mathrm{CDD}}_{i}$$

(2) $${\mathrm{CDD}}_{i}=\frac{1}{24}\overset{24}{\underset{i=1}{\sum }}{\mathrm{CD}}_{i}$$

(3) $${\mathrm{CD}}_{i}=\left\{\begin{array}{c} 0，T_{t}>T_{h}\\ T_{h}-T_{t}，T_{t}\leq T_{h} \end{array}\right.$$

where CDD*_i_* is the cold degree days of the *i*th day, CD*_i_* is the cold degree days of the *i*th hour of the day, *T_h_* (°C) is the threshold temperature of rice subjected to LTS [which was set at 18°C according to the report of Yoshida and Hara (1977)], and *T_t_* (°C) is the ambient temperature.

The correlation between the quality parameters (total content and accumulation of amino acids, essential amino acids, and non-essential amino acids) and ACDD was analyzed. As climatic conditions and rice growth could differ among the three growing seasons, the relative values of quality parameters were used in the linear regressions between quality parameters and ACDD. The relative values of the grain quality parameters were the relative changes in absolute values under different treatments compared to the corresponding values from the control treatment (T1 treatment) for the same treatment stage and cultivar.

### Statistical Analysis

SPSS 23.0 Software (IBM, Inc., United States) was used to analyze the variance of the data. Data were subjected to analysis of variance (ANOVA), and Duncan’s method was used for multiple comparisons among treatments (*p* < 0.05). Curve fitting of amino acids content and accumulation were conducted with OriginPro 2021 software (OriginLab, Wellesley Hills, MA, United States) and relevant parameters were calculated.

## Results

### Effects of LTS on the Grain Yield of Brown Rice

**Figure 2:**
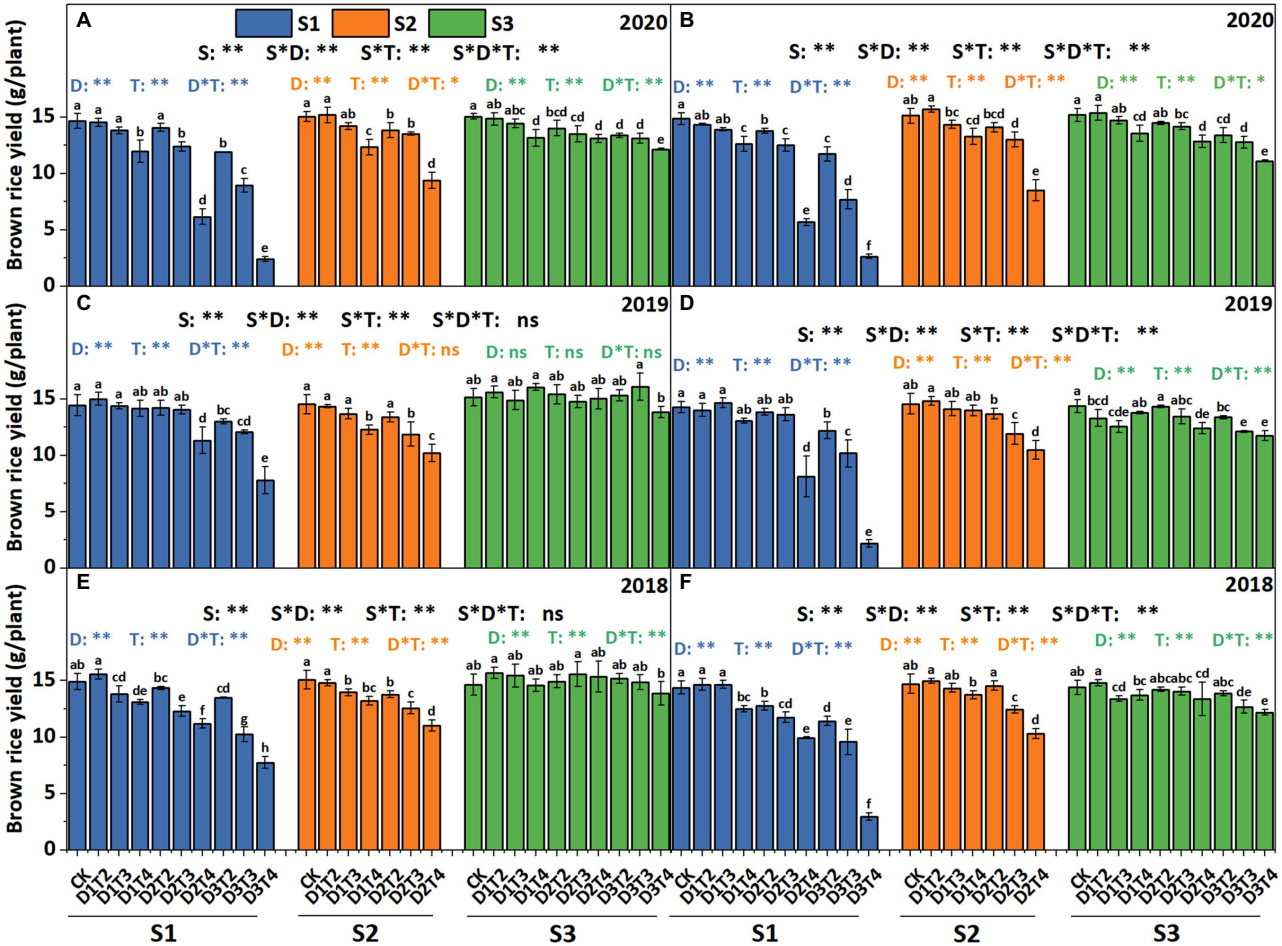
Yield of brown rice under different LTS treatments in Huaidao 5 **(A,C,E)** and Nanjing 46 **(B,D,F)** at maturity. S1, S2, and S3 represent the early stage of flowering, peak stage of flowering, and grain filling stage, respectively. CK indicates the control. The bars with the same lowercase letter(s) indicate no significant differences among different treatments at *p* < 0.05. ^*^ and ^**^ indicate significant effects at *p* < 0.05 and *p* < 0.01 levels, respectively.

As presented in Figure 2, the brown rice yield ranged from 2.16 to 16.10 g plant^−1^. The effect of LTS after flowering on the reduction of brown rice yield was significant, and the rate of reduction varied between different temperature levels, LTS durations and varieties. With the increasing intensity and duration of LTS, the yield of brown rice was gradually declined. For example, the brown rice yield reduction was 3.85, 12.31, and 19.32% under D1, D2, and D3 treatments of Huaidao 5 averaged over the three growing seasons. The average reduction of brown rice yield under D1, D2, and D3 treatment was 2.23, 15.45, and 29.99% of Nanjing 46. The biggest reduction in brown rice yield was under D3T4 treatment in 2020, which was decreased by 83.82 and 82.25% in Huaidao 5 and Nanjing 46, respectively.

In general, the variations in brown rice yield under LTS in Nanjing 46 were larger than those in Huaidao 5, and the brown rice yield showed larger variations under LTS at the early flowering stage than at the other two stages in the two varieties.

### Effects of LTS on Essential Amino Acids

#### Essential Amino Acids Content

There was a pronounced variations in the content of essential amino acids under LTS after flowering, which ranged from −70 to 80% (Figure 2; Supplementary Figures S2, S3). Among the three growing seasons, there were significant interactive effects of LTS at different treatment stages, durations, and temperature levels on essential amino acids content (Table 2).

**Table 2:** ANOVA results for the content of essential amino acids under low-temperature treatments.

Year	Source of variation	Thr	Val	Met	Ile	Leu	Phe	Lys	EAA
2018	Variety (V)	**	**	**	**	**	ns	**	ns
	Stage (S)	**	**	**	**	**	**	**	**
	Duration (D)	**	**	**	**	**	**	ns	**
	Temperature (T)	**	**	**	**	**	**	**	**
	V*S	**	**	**	**	**	**	**	**
	V*D	**	**	**	**	**	**	**	**
	V*T	**	**	ns	**	**	**	**	**
	S*D	**	**	**	**	**	**	*	**
	S*T	**	**	**	**	**	**	**	**
	D*T	**	**	*	**	ns	ns	ns	*
	V*S*D	**	**	**	**	**	**	**	**
	V*S*T	**	**	**	**	**	**	**	**
	V*D*T	**	**	ns	**	**	ns	ns	**
	S*D*T	ns	**	*	*	**	ns	ns	*
	V*S*D*T	ns	**	ns	*	**	*	ns	**
2019	Variety (V)	**	**	**	**	**	**	**	**
	Stage (S)	**	**	**	**	**	**	**	**
	Duration (D)	**	ns	**	**	**	**	**	ns
	Temperature (T)	**	**	**	**	**	*	ns	**
	V*S	**	*	**	**	**	**	**	**
	V*D	**	**	**	**	ns	ns	ns	**
	V*T	**	**	**	**	**	**	ns	*
	S*D	**	**	**	**	**	**	*	**
	S*T	**	**	**	**	**	**	**	**
	D*T	*	ns	ns	**	ns	ns	ns	ns
	V*S*D	**	**	**	**	**	ns	*	*
	V*S*T	**	**	**	**	**	ns	ns	**
	V*D*T	*	ns	ns	*	*	ns	ns	*
	S*D*T	*	ns	ns	**	**	ns	ns	ns
	V*S*D*T	**	**	ns	ns	**	ns	ns	ns
	Variety (V)	**	**	**	**	ns	**	**	**
2020	Stage (S)	**	**	**	**	**	**	**	**
	Duration (D)	**	**	ns	**	ns	**	ns	**
	Temperature (T)	**	**	**	**	**	*	**	**
	V*S	**	**	ns	**	**	**	**	**
	V*D	**	**	*	ns	ns	*	ns	ns
	V*T	**	*	ns	**	ns	ns	*	ns
	S*D	**	**	ns	**	**	ns	**	**
	S*T	**	**	ns	**	**	**	**	**
	D*T	**	*	ns	**	ns	ns	ns	*
	V*S*D	**	**	**	*	ns	ns	ns	**
	V*S*T	**	**	ns	**	ns	*	*	**
	V*D*T	**	ns	ns	ns	ns	ns	ns	ns
	S*D*T	ns	ns	ns	*	ns	ns	ns	**
	V*S*D*T	ns	*	ns	ns	ns	ns	ns	ns

Thr, threonine; Val, valine; Met, methionine; Ile, isoleucine; Leu, leucine; Phe, phenylalanine; Lys, lysine; EAA, total essential amino acids. * *and* ** indicate significant effects at *p*<0.05 and *p*<0.01 levels, respectively.

The Lys, Phe, Leu, and total essential amino acid content showed the same response patterns for the two varieties under LTS (Figure 2; Supplementary Figures S2, S3). In most treatments, the Lys and Phe contents increased under LTS at the early and peak flowering stages but decreased at the grain filling stage in both varieties, and only the Leu content increased at all three stages after flowering.

**Figure 3:**
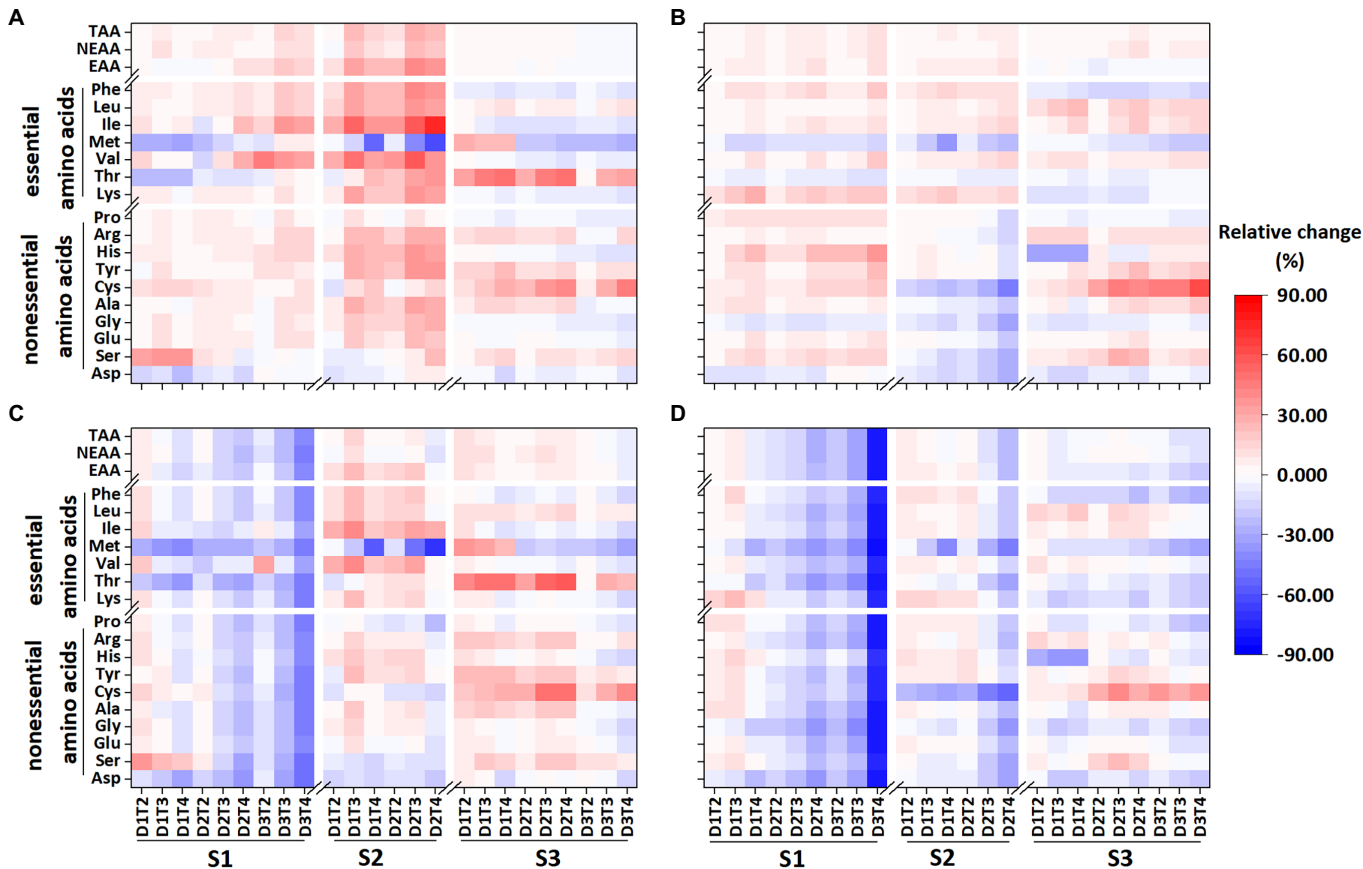
Relative changes in amino acids content **(A,B)** and accumulation **(C,D)** under low-temperature stress in Huaidao 5 **(A,C)** and Nanjing 46 **(B,D)** at maturity in 2018. S1, S2, and S3 represent the early stage of flowering, peak stage of flowering, and grain filling stage, respectively. Thr: threonine, Val: valine, Met: methionine, Ile: isoleucine, Leu: leucine, Phe: phenylalanine, Asp: aspartic acid, Lys: lysine, Ser: serine, Glu: glutamic acid, Gly: glycine, Ala: Alanine, Cys: cysteine, Tyr: tyrosine, His: histidine, Arg: arginine, Pro: proline, EAA: total essential amino acids, NEAA: total non-essential amino acids, TAA: total amino acids. The results for 2019 and 2020 are shown in Supplementary Figures S2, S3.

For Huaidao 5, the content of Met and Thr was increased and then was decreased with increasing the LTS duration at the early flowering stage (Figure 3; Supplementary Figures S2, S3). The Met content decreased at the peak flowering stage and at the grain filling stage with long LTS durations (D2 and D3). The Thr content in Huaidao 5 increased during the peak flowering and grain-filling stages. In Nanjing 46, the Met and Thr contents tended to decrease under LTS in the three stages. The contents of Ile and Val in Huaidao 5 showed an increasing trend at the early and peak flowering stages, but a slightly decreasing trend was observed at the grain-filling stage. In Nanjing 46, the Ile and Val contents showed an increasing trend at all three stages.

Among the seven essential amino acids, Met was the most sensitive to LTS. The total content of essential amino acids showed a larger variation under LTS at the peak flowering stage than at the early flowering and grain-filling stages. Comparing the two varieties, the amino acid content in Huaidao 5 showed a larger variation under LTS than Nanjing 46.

#### Essential Amino Acids Accumulation

According to the analysis of variance (ANOVA; Table 3), the accumulation of most of the essential amino acids was significantly affected by the difference in varieties, treatment stages, low-temperature durations, temperature levels, and their interactions.

**Table 3:** ANOVA results for the accumulation of essential amino acids under low-temperature treatments.

Year	Source of variation	Thr	Val	Met	Ile	Leu	Phe	Lys	EAA
2018	Variety (V)	**	**	**	**	**	**	**	**
	Stage (S)	**	**	**	**	**	**	**	**
	Duration (D)	**	**	**	**	**	**	**	**
	Temperature (T)	**	**	**	**	**	**	**	**
	V*S	**	**	**	**	**	**	**	**
	V*D	**	**	**	**	**	**	**	**
	V*T	**	ns	**	ns	**	*	ns	**
	S*D	ns	**	ns	**	**	**	**	**
	S*T	**	**	**	**	**	**	**	**
	D*T	**	**	**	**	**	**	**	**
	V*S*D	**	**	**	**	**	**	**	**
	V*S*T	**	**	**	**	**	**	**	**
	V*D*T	ns	*	*	**	**	ns	*	*
	S*D*T	**	**	**	**	**	**	**	**
	V*S*D*T	ns	*	ns	ns	ns	*	ns	ns
2019	Variety (V)	**	**	**	**	**	**	**	*
	Stage (S)	**	**	**	**	**	**	**	**
	Duration (D)	**	**	**	**	**	**	**	**
	Temperature (T)	**	**	**	**	**	**	**	**
	V*S	**	**	**	**	**	**	**	**
	V*D	**	**	**	**	**	**	ns	**
	V*T	**	*	**	*	**	**	ns	**
	S*D	**	**	**	**	**	**	**	**
	S*T	**	**	**	**	**	**	**	**
	D*T	**	**	**	**	**	**	**	**
	V*S*D	**	ns	**	*	**	*	ns	**
	V*S*T	**	**	**	**	**	**	*	**
	V*D*T	**	*	**	**	*	ns	ns	ns
	S*D*T	*	**	**	**	**	**	**	**
	V*S*D*T	*	ns	**	*	**	ns	ns	ns
	Variety (V)	**	**	**	**	ns	**	**	**
2020	Stage (S)	**	**	**	**	**	**	**	**
	Duration (D)	**	**	**	**	**	**	**	**
	Temperature (T)	**	**	**	**	**	**	**	**
	V*S	**	**	ns	**	**	**	**	**
	V*D	**	**	**	*	*	**	*	**
	V*T	**	*	**	ns	ns	*	ns	ns
	S*D	**	**	**	**	**	**	**	**
	S*T	**	**	**	**	**	**	**	**
	D*T	**	**	**	**	**	**	**	**
	V*S*D	**	ns	**	**	ns	ns	ns	ns
	V*S*T	**	*	**	**	*	*	*	*
	V*D*T	ns	ns	**	**	ns	ns	*	ns
	S*D*T	**	**	**	**	**	**	**	**
	V*S*D*T	ns	ns	**	ns	ns	ns	ns	ns

Thr, threonine; Val, valine; Met, methionine; Ile, isoleucine; Leu, leucine; Phe, phenylalanine; Lys, lysine; EAA, total essential amino acids. * *and* ** indicate significant effects at *p* < 0.05 and *p* < 0.01 levels, respectively.

In Huaidao 5, the accumulation of Lys, Leu, Val, Ile, and Phe first increased and then decreased with increasing duration of LTS treatments at the early flowering stage, whereas the accumulation of Met and Thr was decreased under all treatments. At the peak flowering stage, the accumulation of most essential amino acids was increased under most LTS treatments, especially under T4 levels, except for Met, which showed a decreasing trend under LTS (Figure 3; Supplementary Figures S2, S3). At the grain-filling stage, the accumulation of Lys, Phe, Val, Met, and Ile increased with slight LTS treatments and decreased with serve LTS treatments, and the accumulation of Thr increased under all LTS treatments. In Nanjing 46, the accumulation of all essential amino acids decreased under most LTS treatments at the early flowering stage, except some for slight LTS treatments (D1T2 and D1T3). At the peak flowering stage, most essential amino acids accumulation first increased and then decreased with increasing duration of LTS, except for Met and Thr in 2018. However, at the grain-filling stage, LTS slightly decreased Phe, Met, Thr, and Lys accumulation under most LTS treatments.

In general, the variations in essential amino acid accumulation under LTS in Nanjing 46 were more than those in Huaidao 5, and the accumulation of essential amino acids showed larger variations under LTS at the early flowering stage than at the other two stages in Huaidao 5. Among the seven essential amino acids, Met accumulation was the most sensitive to LTS, similar to the content of essential amino acids (Figure 3; Supplementary Figures S2, S3).

### Effects of LTS on Non-essential Amino Acids

#### Non-essential Amino Acids Content

The content of non-essential amino acids varied significantly under LTS, ranging from −65.04 to 60.17% (Figure 3; Supplementary Figures S2, S3). The content of most of the non-essential amino acids showed significant differences among the different varieties, treatment stages, durations, and intensities of LTS (Supplementary Table S1). However, the interaction of low-temperature duration, intensity, and their interaction with treatment stage was not significant for several non-essential amino acids (Supplementary Table S1).

For LTS, at the early flowering (S1) and grain-filling stages (S3), the content of most non-essential amino acids in the two varieties showed similar results. At early flowering (S1), the content of most non-essential amino acids in the two varieties was increased, except for the contents of Asp in Huaidao 5, and Asp and Gly in Nanjing 46, which decreased in all treatments (Figure 3; Supplementary Figures S2, S3). At the grain-filling stage (S3), the content of Pro, His, Gly, and Asp was decreased in the two varieties, while the content of other non-essential amino acids was increased. However, for LTS at the peak flowering stage (S2), the content of non-essential amino acids was generally increased in Huaidao 5 but decreased in Nanjing 46.

In general, the non-essential amino acid content was more sensitive to LTS at the peak flowering stage than at the early flowering and grain-filling stages. Among the ten non-essential amino acids, Cys was the most sensitive to LTS (Figure 3 Supplementary Figures S2, S3). In addition, the non-essential amino acid content in Huaidao 5 was more sensitive to LTS than those in Nanjing 46.

#### Non-essential Amino Acids Accumulation

The results of ANOVA showed that the accumulation of most non-essential amino acids varied significantly in the cultivar, stage, duration, and intensity of LTS (Supplementary Table S2). At the early flowering stage, the accumulation of most non-essential amino acids in Huaidao 5 increased under lightly LTS treatments (D1T2, D1T3, and D2T2) and decreased under D3 duration and D2T4, except for the accumulation of Asp., which decreased in all treatments at the early flowering stage (Figure 3 Supplementary Figures S2, S3). For LTS at the peak flowering, the accumulation of most non-essential amino acids increased under slight LTS treatments in Huaidao 5, except for Asp, Ser in 2018 and Asp, Ala, and Gly in 2019 were decreased in all treatments. The accumulation of non-essential amino acids in Nanjing 46 also showed an increasing trend under lightly LTS treatments (D1T2, D1T3, and D2T2) at the early flowering. At the grain-filling stage, the changing trend of non-essential amino acids accumulation was not consistent for 3 years.

Overall, the accumulation of non-essential amino acids was more sensitive to LTS at the peak flowering stage than at the early flowering and grain-filling stages. Among the two varieties, the accumulation of non-essential amino acids in Nanjing 46 was more sensitive to LTS than that in Huaidao 5 (Figure 3; Supplementary Figures S2, S3). Among the ten non-essential amino acids, Cys accumulation was the most sensitive.

### Quantifying the Effects of LTS on Amino Acids

#### Quantifying the Effects of LTS on Amino Acids Content

**Figure 4:**
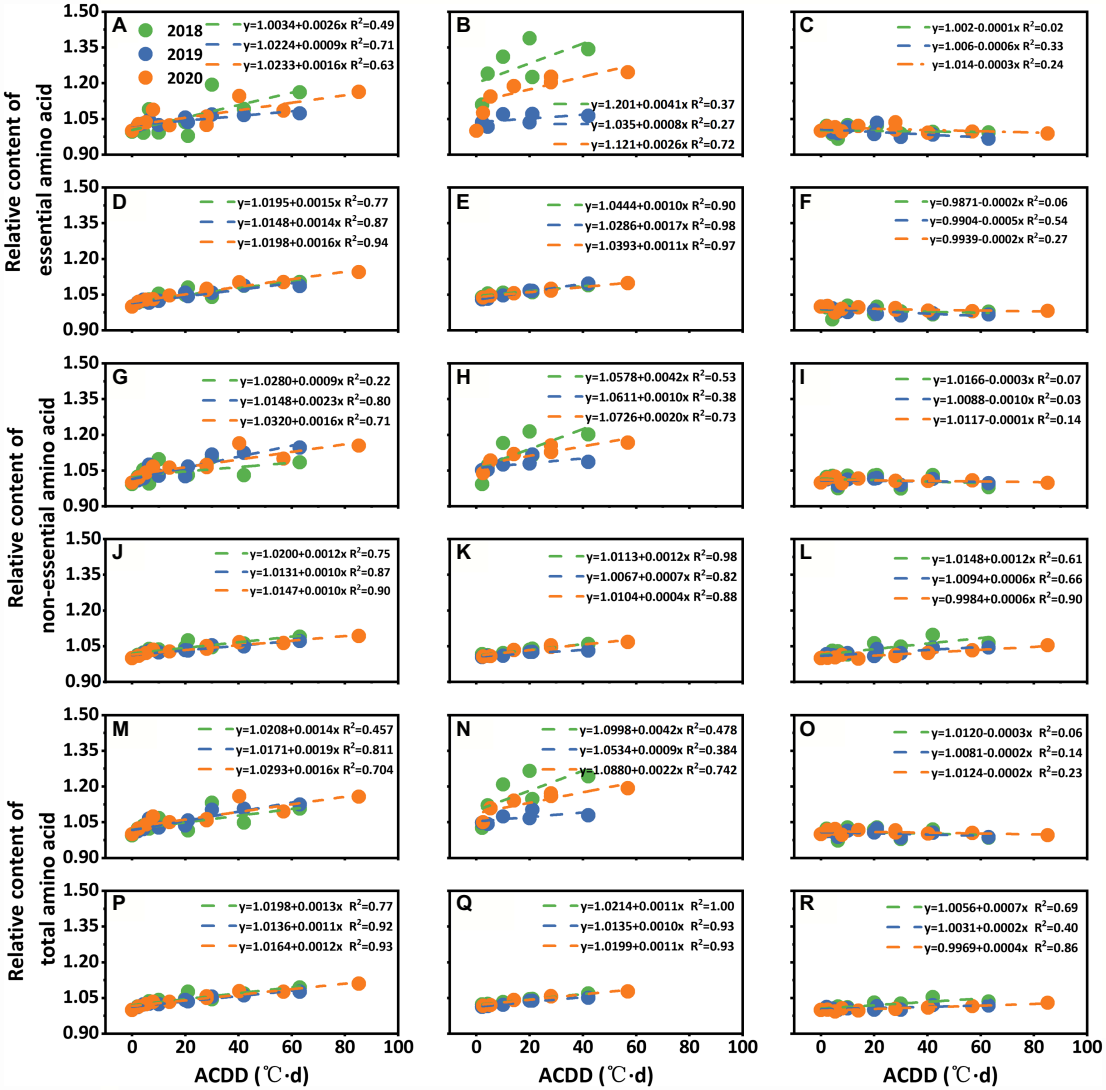
Relationship between low-temperature stress at early flowering **(A,D,G,J,M,P)**, peak flowering **(B,E,H,K,N,Q)**, and grain filling **(C,F,I,L,O,R)** stages and the relative contents of amino acids in Huaidao 5 **(A–C,G–I,M–O)** and Nanjing 46 **(D–F,J–L,P–R)** at maturity. ACDD: accumulated cold degree days. Three different colors indicate results from three growing seasons in 2018–2020. **(A–F)** present the relative content of essential amino acids. **(G–L)** present the relative content of non-essential amino acids. **(M–R)** present the relative content of total amino acids.

According to the fitting results of ACDD and relative amino acids content, the essential, non-essential, and total amino acids content increased with increasing ACDD under LTS at early and peak flowering stages in both varieties (Figure 4). However, under LTS at the grain-filling stage, the changes of amino acids content in the two varieties were relatively small. For example, with a 1°C·d increase in ACDD at the early flowering stage (S1), the relative content of essential amino acid increased by 0.14–0.16% and 0.09–0.26% for Nanjing 46 and Huaidao 5, respectively (Figures 4A,D). For a 1°C d increase in ACDD at the peak flowering stage (S2), the essential amino acid content increased by 0.08–0.041% and 0.10–0.17% for Nanjing 46 and Huaidao 5, respectively (Figures 4B,E). Generally, the effects of LTS at the peak flowering stage (S2) on the essential, non-essential, and total amino acid content were larger than those at early flowering (S1) and grain filling stages (S3).

When comparing the results among the three growing seasons, the interannual differences for the quantified relationships were relatively small, except for the treatments at the peak flowering stage for Huaidao 5 (Figure 4). The content of essential, non-essential, and total amino acids in Huaidao 5 was more sensitive to LTS at early (S1) and peak flowering stages (S2) compared to Nanjing 46, except for treatments in 2019, whereas no significant difference in any of the contents was observed between the two varieties at grain filling stages (S3). In addition, there were no significant differences between the responses of the total essential amino acid and total non-essential amino acid contents under LTS.

#### Quantifying the Effects of LTS on Amino Acids Accumulation

**Figure 5:**
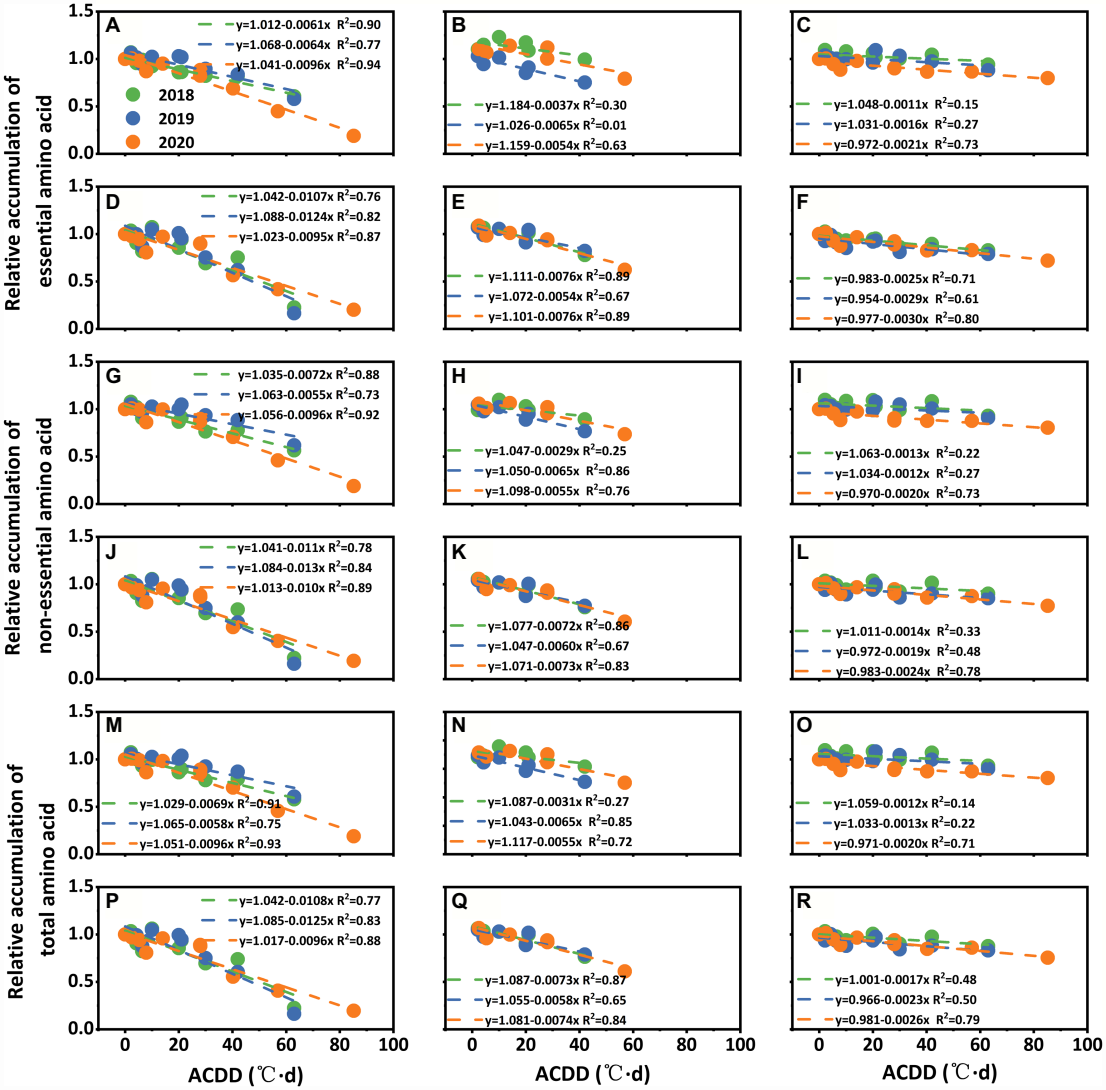
Relationship between low-temperature stress at early flowering **(A,D,G,J,M,P)**, peak flowering **(B,E,H,K,N,Q)**, and grain filling **(C,F,I,L,O,R)** stages and the relative accumulation of amino acids in Huaidao 5 **(A–C,G–I,M–O)** and Nanjing 46 **(D–F,J–L,P–R)** at maturity. ACDD: accumulated cold degree days. Three different colors indicate results from three growing seasons in 2018–2020. **(A–F)** present the relative accumulation of essential amino acids. **(G–L)** present the relative accumulation of non-essential amino acids. **(M–R)** present the relative accumulation of total amino acids.

The accumulation of amino acids was affected by both the amino acid content and rice yield. The fitting results of ACDD and the accumulation of essential, non-essential, and total amino acids showed a same trend at three stages, which decreased with increasing ACDD (Figure 5). However, under LTS at the grain-filling stage, the change in amino acids accumulation between the two varieties was relatively small. For example, with a 1°C·d increase in ACDD at the early flowering stage (S1), the relative accumulation of essential amino acid decreased by 0.95–1.24% and 0.61–0.96% for Nanjing 46 and Huaidao 5, respectively (Figures 5A,D). For a 1°C·d increase in ACDD at the peak flowering (S2), the accumulation of essential amino acid increased by 0.54–0.76% and 0.37–0.65% for Nanjing 46 and Huaidao 5, respectively (Figures 5B,E). Generally, at early flowering (S1), the effects of LTS on the accumulation of essential, non-essential, and total amino acids were larger than those at the peak flowering stage (S2) and grain filling stages (S3).

When comparing the results among the three growing seasons, the interannual differences in the quantified relationships were relatively small. The accumulation of essential, non-essential, and total amino acids in Nanjing 46 showed higher sensitivity to LTS at the early (S1) and peak flowering stages (S2) than Huaidao 5 (Figure 5). Amino acids accumulation showed no obvious differences between the two varieties at the grain-filling stages (S3). The accumulation of total essential amino acids and total non-essential amino acids showed no significant differences under different LTSs.

## Discussion

### Effects of LTS on Amino Acids Content

Global diets are highly reliant on cereals, most particularly rice. Any change in the yield or quality can affect the dietary health of millions of people. Our results from controlled chamber experiments offer robust evidence that the LTS after flowering associated with climate change affects the amino acids content of rice. For most amino acids content, it was shown that the content of amino acids in rice is increased under LTS, which was similar to previous results under other extreme climate events (e.g., heat and shading; Liang et al., 2015; Yang et al., 2016). Surprisingly, Lys content in rice was significantly increased under LTS at the early flowering and peak flowering stages in our study (Figure 3; Supplementary Figures S2, S3). The lack of Lys, an essential amino acid in rice, results in limited food diversity and poses a greater risk of malnutrition to a population relying on rice as the main food (Mir et al., 2018). Therefore, the effect of LTS after flowering could increase the ability of rice to meet adult daily dietary Lys requirements. Furthermore, it was found that the Asp content in rice grains significantly decreased under LTS after flowering, while the Glu content significantly increased.

LTS has been shown to affect the content of several nutrient in rice grains (Jia et al., 2015b; Huang et al., 2021). There were several possible reasons for LTS effects on content of amino acids. On the one hand, the synthesis of amino acids in rice grains mainly depends on the translocation of glutamine from vegetative organs (leaf, leaf sheath, and stem) to the grain (Yang et al., 2020). Previous studies have shown that LTS substantially elevated levels of free aspartate, asparagine and glutamine in rice leaves (Liu et al., 2022). This indicated that LTS may affect the translocation of glutamine from leaves to grains. In addition, LTS could also affect the activity of aminoacyl synthetases in rice grains. In the case of temperature effects on the activities of aspartate metabolism enzymes, Liang et al. (2013) found that higher temperature increased the average activities of aspartate aminotransferase and aspartokinase in rice grains, thus significantly increased the amino acid contents of Asp., Lys, Thr, Met, and Ile and the protein contents of albumin, globulin, prolamin, and glutelin. Here, the activity of aspartate aminotransferase may be notably decreased by LTS, and this may hinder the conversion of Glu to Asp, resulting in an increase in Glu content and a decrease in Asp content. To dissect the exact mechanisms for this, measuring the glutamine content in the vegetative organs and the activity of aminoacyl synthetases in the grains under LTS will be needed in the future research.

Furthermore, the increase in amino acid content was also due to the dilution effect caused by decreasing grain yield under LTS at the flowering stage. Our previous results with this same experiment showed that LTS after flowering did not significantly decrease grain weight, except under D3T4 treatment in Nanjing 46 (Ali et al., 2021). And this result was similar to the previous studies (Huang et al., 2021; Shi et al., 2021). Therefore, we assume that the main reason for the dilution effect of the amino acids content could be the decreasing grain number due to reduced seed-setting rate under LTS, as reported in our previous study (Ali et al., 2021), and the decreasing grain number under LTS could significantly reduce the sink capacity. Moreover, the nitrogen uptake of rice under LTS maintains to a larger extent (Jia et al., 2015a). Therefore, we assume that the nitrogen sources mostly from green leaves for amino acids synthesis in grain were relatively abundant, and this have been observed under extreme heat stress (Zhen et al., 2020). So, the reduced sink capacity in the grain and the relative surplus of nitrogen sources could result in higher amino acid accumulation per grain to some extent, and eventually increasing the content of some amino acids. This phenomenon has also been reported in nitrogen accumulation study under low water temperature during reproductive period (Jia et al., 2019).

### Effects of LTS on Amino Acids Accumulation

Amino acid accumulation was mainly determined by both amino acid content and yield of brown rice. Temperature is a major determinant for growth and yield of rice, LTS has effects on rice growth and yield, among which yield and spikelet sterility can play pivotal roles (Jia et al., 2015b). Our results showed that the brown rice yield of most LTS treatments after flowering significantly decreased in the three-growing season, compared to the control. But, the accumulation of amino acids in rice grains will be very different under LTS at different stages after flowering (Figure 3; Supplementary Figures S2, S3). Although the content of some amino acids was significantly increased during the early and peak flowering stages, their accumulation was significantly decreased. The accumulation of amino acids showed larger variations under LTS at the early flowering stage than at the other two stages. Some previous research documented that spikelet fertility, grain filling and seed setting rates were mainly influenced during the last week of pre-heading to heading (Siddik et al., 2019). Therefore, proper change of phenological development through cultivation and management measures to avoid LTS at the early flowering stage can help to reduce the impact of LTS on crop yield and quality. Andrianary et al. (2021) has reported that phosphorus application affects lowland rice yields by changing phenological development and reducing cold stress events in the central highlands of Madagascar. In addition, previous studies also have shown that fertilization and irrigation are the two main cultivation practices that regulate rice yield and quality (Huang et al., 2019b; Haghighi et al., 2020). However, whether these methods can alleviate the effects of LTS on amino acids in grains remains to be explored.

### Differences in the Responses of Amino Acids to LTS Among Rice Varieties

In order to explore whether improving LTS tolerance of rice varieties can effectively alleviate the impact of LTS on yield and quality, we used two rice varieties with different tolerance to LTS, namely, Huaidao 5 (tolerant to LTS) and Nanjing 46 (sensitive to LTS), to systematically investigated the responses of the brown rice yield and amino acids quality under LTS. In this study, the amino acid content in Huaidao 5 was more sensitive than Nanjing 46 under LTS, but the results for the accumulation of amino acids and brown rice yield was opposite, which may be due to the different stress tolerance of grain yield and amino acid content to LTS between the two varieties. Plants stress tolerance is closely related to the amino acid content (Ashraf and Foolad, 2007). Amino acids can serve as signaling molecules that regulate the structural composition of roots, stems, and flowering time and resist biotic and abiotic stresses. Larosa et al. (1991) showed that the accumulation of Pro is positively correlated with plant stress tolerance. Moreover, the content and accumulation of Pro in plants increased under abiotic stress, which is consistent with the results of this study under LTS at the early and peak flowering stages (Figure 3; Supplementary Figures S2, S3).

The variation of brown rice yield and amino acids quality under LTS across the two rice varieties suggested a notably genotypical differences between varieties, and this offer a good opportunity for breeding towards more climate-resilient and could partly address these new challenges to global health (Myers et al., 2014). For improving LTS tolerance in rice, large numbers of studies have been conducted through genome editing tools and artificial intelligence technology recently (Zhu, 2016; Zhang et al., 2022).

### Quantifying the Effects of LTS on Amino Acids

There were several studies focused on the impacts of environmental conditions on amino acids of rice, but most of them were qualitative and did not quantify the variation of amino acids in different environments (Huang et al., 2019a; Guo et al., 2021). In this study, ACDD was used to quantify the effects of different LTS intensities and durations on the content and accumulation of amino acids at three stages (Figures 4, 5), supporting earlier observations and modelling. The results showed that the amino acid content varied among different growing seasons, but the relative responses of amino acids content to LTS were similar among the three growing seasons, except for the treatments in Huaidao 5 at the peak flowering stage. In recent years, process-based crop growth models, which provide an implementation of crop physiological growth process and its interactions with genotype, soil, management, and weather conditions, have been widely used to quantify the impacts of climate change on crop production (Liu et al., 2018; Sun et al., 2021). However, most of the previous studies focused on grain yield (Sun et al., 2018), while a few studies on grain quality have focused solely on protein and starch (Osman et al., 2020, 2021), and the simulation or prediction of amino acid variation under climate change has not been reported. Thus, most of the previous studies on climate change impact assessment focused solely on food security in terms of food quantity, without focusing on the food nutritional quality. The quantitative results of this study will provide a strong basis for simulating the impact of climate change on amino acid content in future crop models.

## Conclusion

In summary, the effects of LTS on amino acid content and accumulation in rice grains at maturity were systematically quantified. LTS had significant interactive effects at different treatment stages, durations, and temperature levels on the content and accumulation of amino acids. Generally, LTS increased the essential, non-essential, and total amino acid content in the two rice varieties but decreased the accumulation of these amino acids. Among the seven essential amino acids, the content of Met was the most sensitive to LTS, while among the ten non-essential amino acids, the content of Cys was the most sensitive to LTS. In addition, the amino acid content in Huaidao 5 was more sensitive to LTS after flowering, while the accumulation of amino acids in Nanjing 46 was more sensitive than that in Huaidao 5. The relative content of essential amino acids increased by 0.14–0.16% and 0.09–0.26% with a 1°C·d increase in ACDD at early flowering (S1) for Nanjing 46 and Huaidao 5, respectively. And the accumulation of essential amino acids decreased by 0.95–1.24% and 0.61–0.96% with a 1°C·d increase in ACDD at early flowering (S1) for Nanjing 46 and Huaidao 5, respectively. The results of this study suggest that LTS at flowering stage can improve nutritional quality of amino acids in rice, while decreasing its accumulation, with higher sensitivities to LTS at the flowering stage than at the grain filling stage.

## Data Availability Statement

The original contributions presented in the study are included in the article/Supplementary Material, further inquiries can be directed to the corresponding author.

## Author Contributions

BL, YZ, and LT designed and supervised the project. MK, GL, YZ, JS, and JZ performed the environment-controlled phytotron experiments and analyzed the data. BL and LL provided critical feedback and helped to shape the research. MK and GL wrote the manuscript with help from BL, YZ, and LT for critical revisions and drafting. All authors contributed to the article and approved the submitted version.

## Funding

This research was funded by the National Key Research and Development Program of China (2019YFA0607404), the National Science Foundation for Distinguished Young Scholars (31725020), and the National Science Foundation of China (32021004, and 41961124008).

## Conflict of Interest

The authors declare that the research was conducted in the absence of any commercial or financial relationships that could be construed as a potential conflict of interest.

## Publisher’s Note

All claims expressed in this article are solely those of the authors and do not necessarily represent those of their affiliated organizations, or those of the publisher, the editors and the reviewers. Any product that may be evaluated in this article, or claim that may be made by its manufacturer, is not guaranteed or endorsed by the publisher.
