# Supplementary material for: Extreme Low-Temperature Stress Affects Nutritional Quality of Amino Acids in Rice
Source: Front Plant Sci. 2022 Jun 2;13:905348. doi: 10.3389/fpls.2022.905348 (PMC9201379; doi:10.3389/fpls.2022.905348)
Supplement: Supplementary file 1 [file Data_Sheet_1.docx]

Supplementary Material

**Supplementary Table 1. ANOVA results for the content of non-essential amino acid under low-temperature treatments.**

| Year | Source of variation | Asp | Ser | Glu | Gly | Ala | Cys | Tyr | His | |
| --- | --- | --- | --- | --- | --- | --- | --- | --- | --- | --- |
| 2018 | Variety (V) | ** | ** | ** | ** | ** | ns | ** | ** | |
|  | Stage (S) | ** | ** | ** | ** | ** | ** | ** | ** | |
|  | Duration (D) | * | ** | * | ** | ** | ** | ns | ** | |
|  | Temperature (T) | ns | * | ns | ** | ** | ** | ** | ** | |
|  | V*S | ** | ** | ** | ** | ** | ** | ** | ** | |
|  | V*D | ** | ** | ** | ** | ** | ** | ** | ** | |
|  | V*T | ** | ** | ** | ns | ** | ** | ** | ** | |
|  | S*D | ** | ** | ns | ** | ** | ** | ** | * | |
|  | S*T | ** | ** | ** | ** | ** | ** | ** | ** | |
|  | D*T | ** | ns | ns | ** | * | ns | ** | ** | |
|  | V*S*D | ns | ** | * | ns | ** | ** | ** | ** | |
|  | V*S*T | ** | ** | ** | ** | ** | ** | ** | ** | |
|  | V*D*T | ** | ** | ** | ** | ** | ns | ** | ** | |
|  | S*D*T | ** | ** | ** | ** | ** | * | ** | ** | |
|  | V*S*D*T | ** | ** | ** | ** | ** | ** | ** | ** | |
| 2019 | Variety (V) | ** | ** | ** | ** | ** | ** | ** | ** | |
|  | Stage (S) | ** | ** | ** | ** | ** | ** | ** | ** | |
|  | Duration (D) | ns | ns | * | ns | ** | ** | ns | ** | |
|  | Temperature (T) | ** | ** | ** | * | ns | ns | ns | ** | |
|  | V*S | ** | ** | ** | ** | ** | ** | ** | ** | |
|  | V*D | ** | ** | ns | * | ns | ** | ** | ** | |
|  | V*T | ** | ** | ** | ns | ** | ** | ** | * | |
|  | S*D | * | ** | ** | ** | ** | ** | ns | ** | |
|  | S*T | ** | ** | ** | ns | ** | ** | ** | ** | |
|  | D*T | * | ** | ns | * | * | ** | ns | ** | |
|  | V*S*D | ns | ** | ** | ns | ** | ** | ** | ** | |
|  | V*S*T | ** | ** | ** | ns | ** | ** | ** | ** | |
|  | V*D*T | ** | ** | * | ** | * | ** | ** | * | |
|  | S*D*T | ns | ** | ** | ns | ** | ** | ** | ns | |
|  | V*S*D*T | * | ** | ns | ** | ** | ** | ** | * | |
| 2020 | Variety (V) | ** | ** | ** | ** | ** | ** | ** | ** | |
|  | Stage (S) | ** | ** | ** | ** | ** | ** | ns | ** |  |
|  | Duration (D) | ns | ** | ** | ns | ** | ** | ns | ** |  |
|  | Temperature (T) | * | ** | ** | * | ** | ** | ** | ** |  |
|  | V*S | * | ** | ** | ** | ** | ** | ** | ** |  |
|  | V*D | ns | ns | ns | ns | ** | ** | ns | ** |  |
|  | V*T | ns | ** | ns | ** | ** | ns | ns | ns |  |
|  | S*D | * | ns | ns | ns | ** | ** | ** | ** |  |
|  | S*T | ns | * | ** | ** | ** | ** | ns | ** |  |
|  | D*T | ns | ns | ns | ns | ** | ns | ns | ns |  |
|  | V*S*D | * | ** | ** | ** | ** | ** | ns | ns |  |
|  | V*S*T | ns | * | * | * | ** | ns | ns | ns |  |
|  | V*D*T | ns | ns | ns | ns | ** | ns | ns | ns |  |
|  | S*D*T | ns | ns | ns | ns | ** | * | ns | ns |  |
|  | V*S*D*T | ns | ns | ns | ns | ** | ns | ns | ns |  |

Note: Ser: serine, Glu: glutamic acid, Gly: glycine, Ala: Alanine, Cys: cystine, Asp: aspartic acid, Tyr: tyrosine, His: histidine, Arg: arginine, Pro: proline, NEAA: the total of non-essential amino acids. * and ** means significant effects at p＜0.05 and p＜0.01 levels, respectively.

**Supplementary Table 2. ANOVA results for the accumulation of non-essential amino acid under low-temperature treatments.**

| Year | Source of variation | Asp | Ser | Glu | Gly | Ala | Cys | Tyr | His | |
| --- | --- | --- | --- | --- | --- | --- | --- | --- | --- | --- |
| 2018 | Variety (V) | ** | * | ** | ** | ** | ** | ns | ** | |
|  | Stage (S) | ** | ** | ** | ** | ** | ** | ** | ** | |
|  | Duration (D) | ** | ** | ** | ** | ** | ** | ** | ** | |
|  | Temperature (T) | ** | ** | ** | ** | ** | ** | ** | ** | |
|  | V*S | ** | ns | ** | ** | ** | ** | ** | ** | |
|  | V*D | ** | ns | ** | ns | * | ns | ns | * | |
|  | V*T | ** | ** | ns | ** | ** | ** | ** | ** | |
|  | S*D | ** | ** | ** | ** | ** | ** | ** | ** | |
|  | S*T | ** | ** | ** | ** | ** | ** | ** | ** | |
|  | D*T | ** | ** | ** | ** | ** | ** | ** | ** | |
|  | V*S*D | ** | ** | ** | ** | ** | ** | ** | ** | |
|  | V*S*T | ** | * | ** | ** | ** | ** | ** | ** | |
|  | V*D*T | ** | ns | * | ns | ns | * | ns | ns | |
|  | S*D*T | ** | * | ** | ** | ** | ** | ** | ** | |
|  | V*S*D*T | * | * | ns | ns | ** | ns | ns | ns | |
| 2019 | Variety (V) | ** | ** | ** | ** | ** | * | * | ** | |
|  | Stage (S) | ** | ** | ** | ** | ** | ** | ** | ** | |
|  | Duration (D) | ** | ** | ** | ** | ** | ** | ** | ** | |
|  | Temperature (T) | ** | ** | ** | ** | ** | ** | ** | ** | |
|  | V*S | * | ** | ** | ** | ** | ** | ** | ** | |
|  | V*D | ** | * | ** | ** | ** | ** | * | ns | |
|  | V*T | ** | ** | ** | ** | ** | ** | ** | ** | |
|  | S*D | ** | ** | ** | ** | ** | ** | ** | ** | |
|  | S*T | ** | ** | ** | ** | ** | ** | ** | ** | |
|  | D*T | ** | ** | ** | ** | ** | ** | ** | ** | |
|  | V*S*D | ** | ** | ** | ns | ns | ** | ** | ** | |
|  | V*S*T | ** | * | ** | ** | ** | ** | ** | ** | |
|  | V*D*T | ** | ns | * | ns | ** | ns | ns | ns | |
|  | S*D*T | ** | ** | ** | ** | ** | ns | ** | ** | |
|  | V*S*D*T | ** | ns | ns | ns | * | * | * | * | |
| 2020 | Variety (V) | ** | ** | ** | ** | ** | ** | ** | ** | |
|  | Stage (S) | ** | ** | ** | ** | ** | ** | ** | ** |  |
|  | Duration (D) | ** | ** | ** | ** | ** | ** | ** | ** |  |
|  | Temperature (T) | ** | ** | ** | ** | ** | ** | ** | ** |  |
|  | V*S | ** | ns | ** | ** | ** | ** | ** | ** |  |
|  | V*D | ** | ns | ns | ns | ** | * | ** | ns |  |
|  | V*T | ** | ns | ns | * | ** | ** | ** | ns |  |
|  | S*D | ** | ** | ** | ** | ** | ** | ** | ** |  |
|  | S*T | ** | ** | ** | ** | ** | ** | ** | ** |  |
|  | D*T | ** | ** | ** | ** | ** | ** | ** | ** |  |
|  | V*S*D | ns | ** | ns | ns | ** | ** | ** | ns |  |
|  | V*S*T | ns | * | ns | ns | * | ns | ns | ns |  |
|  | V*D*T | * | ns | ns | ns | ** | * | ** | ns |  |
|  | S*D*T | ** | ** | ** | ** | ** | ** | ** | ** |  |
|  | V*S*D*T | ns | ns | ns | ns | ** | ns | ns | ns |  |

Note: Ser: serine, Glu: glutamic acid, Gly: glycine, Ala: Alanine, Cys: cystine, Asp: aspartic acid, Tyr: tyrosine, His: histidine, Arg: arginine, Pro: proline, NEAA: the total of non-essential amino acids. * and ** means significant effects at p＜0.05 and p＜0.01 levels, respectively.


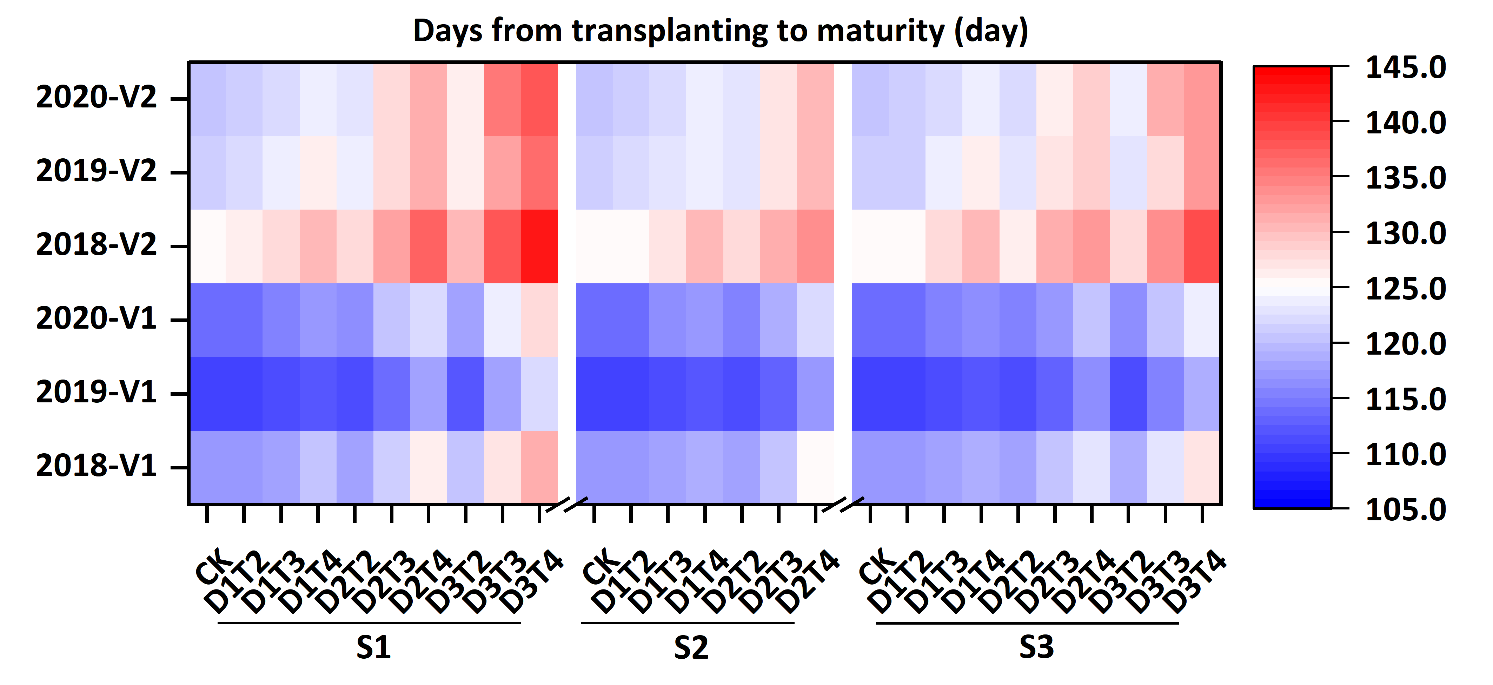


**Supplementary Figure 1. Days from transplanting to maturity for each treatment under LTS after flowering from three growing seasons in 2018–2020. V1** and **V2** represents the Huaidao 5 and Nanjing 46, respectively. **CK** indicate the control. **S1**, **S2**, and **S3** represents the early stage of flowering, peak stage of flowering, and grain filling stage, respectively.


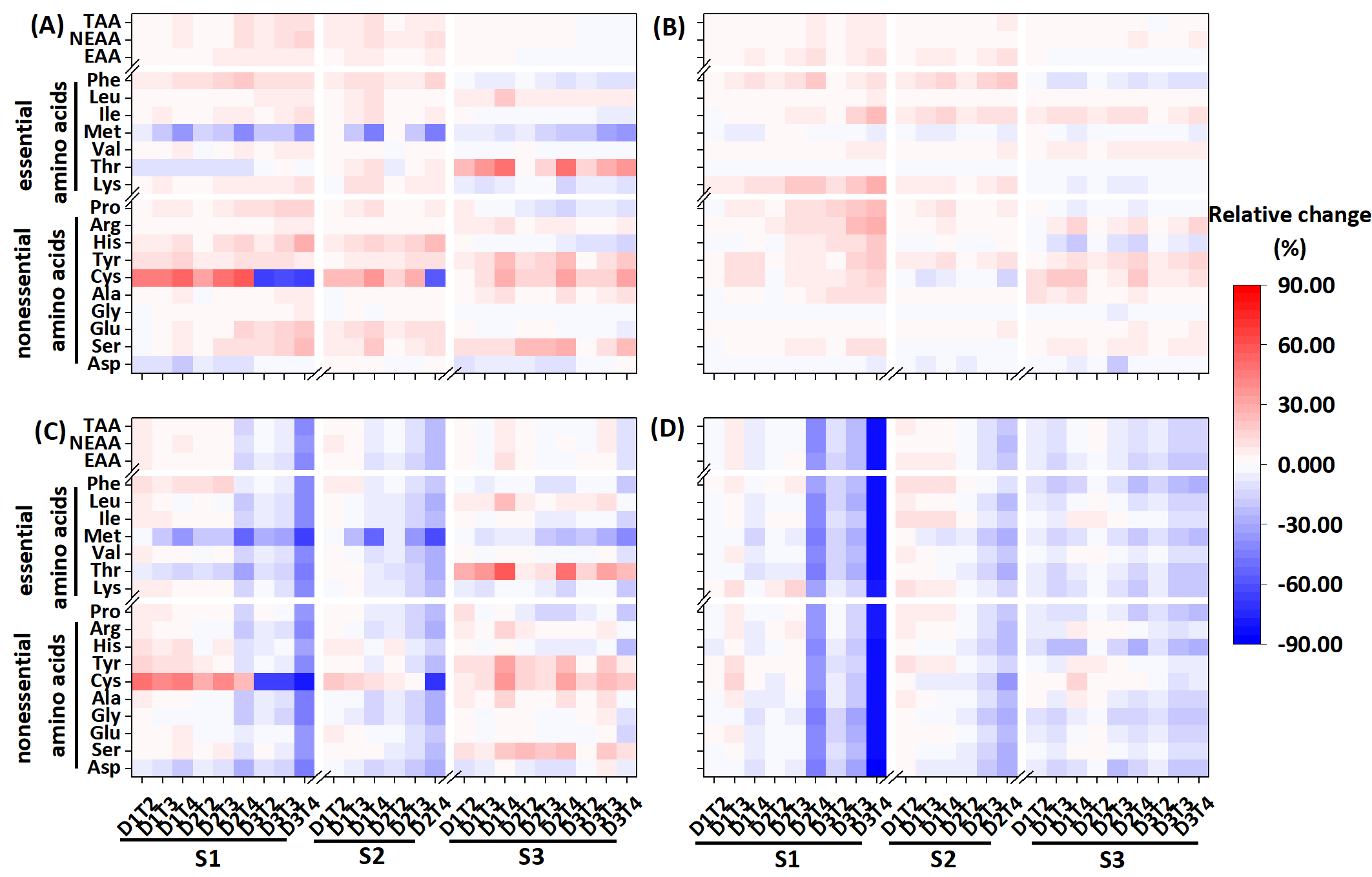


**Supplementary Figure 2. Relative changes of amino acid content (a, b) and accumulation (c, d) under low-temperature stress in Huaidao 5 (a, c) and Nanjing 46 (b, d) at maturity in 2019.** **S1**, **S2**, and **S3** represent the early stage of flowering, peak stage of flowering, and grain filling stage, respectively. **Thr**: threonine, **Val**: valine, **Met**: methionine, **Ile**: isoleucine, **Leu**: leucine, **Phe**: phenylalanine, **Asp**: aspartic acid, **Lys**: lysine, **Ser**: serine, **Glu**: glutamic acid, **Gly**: glycine, **Ala**: Alanine, **Cys**: cysteine, **Tyr**: tyrosine, **His**: histidine, **Arg**: arginine, **Pro**: proline, **EAA**: total essential amino acids, **NEAA**: total non-essential amino acids, **TAA**: total amino acids.


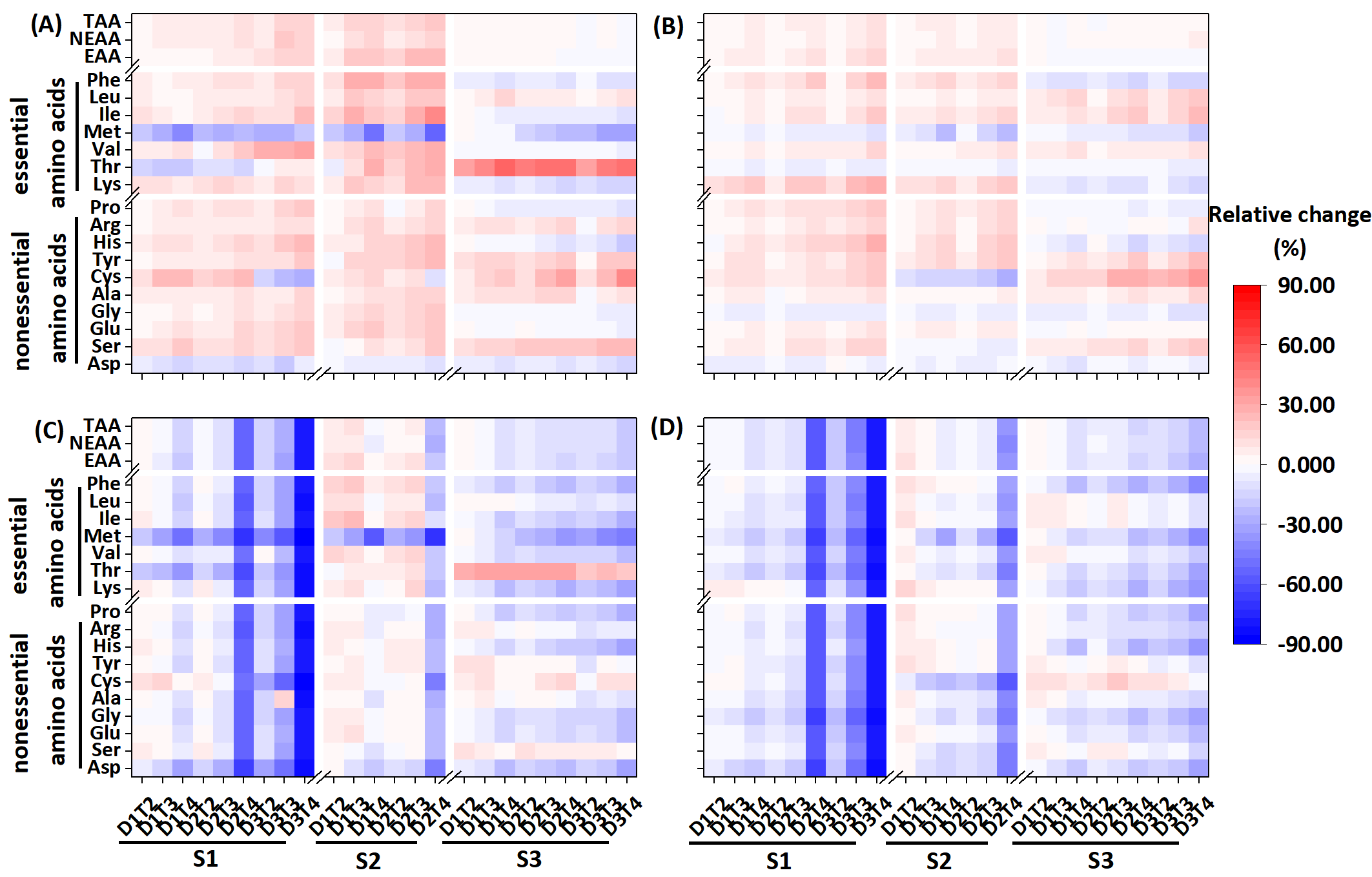


**Supplementary Figure 3. Relative changes of amino acid content (a, b) and accumulation (c, d) under low-temperature stress in Huaidao 5 (a, c) and Nanjing 46 (b, d) at maturity in 2020. S1**, **S2**, and **S3** represent the early stage of flowering, peak stage of flowering, and grain filling stage, respectively. **Thr**: threonine, **Val**: valine, **Met**: methionine, **Ile**: isoleucine, **Leu**: leucine, **Phe**: phenylalanine, **Asp**: aspartic acid, **Lys**: lysine, **Ser**: serine, **Glu**: glutamic acid, **Gly**: glycine, **Ala**: Alanine, **Cys**: cysteine, **Tyr**: tyrosine, **His**: histidine, **Arg**: arginine, **Pro**: proline, **EAA**: total essential amino acids, **NEAA**: total non-essential amino acids, **TAA**: total amino acids.
